# Hematological parameters in champion of Brazilian jiu‐jitsu paradesport: Case study

**DOI:** 10.14814/phy2.14435

**Published:** 2020-06-17

**Authors:** Jaqueline S. S. Lopes, Aníbal M. de Magalhães Neto, Aline C. de Almeida, Paulo R. L. Alves, Elcirley L. Silva, Márcio V. de Abreu Verli, Claudia M. B. Andrade

**Affiliations:** ^1^ Federal University of Mato Grosso (UFTM) Postgraduate Program in Health Sciences (PPGSC) Cuiabá MT Brazil; ^2^ Departament of Physical Therapy Federal University of São Carlos (UFSCAR) São Carlos SP Brazil; ^3^ Fundação de Apoio à Escola Técnica Escola Técnica Estadual Visconde de Mauá Rio de Janeiro ‐ RJ Brazil

**Keywords:** combat sport, disabled athletes, martial arts, muscle damage, sports performance

## Abstract

The behavior of biochemical and immunological parameters investigated in the field conditions in athletes is important to influence in the management of recovery and disease prevention as well as, to support the training program, as well as to improve the physical conditioning associated with health and performance. However, for amputee athletes, Brazilian jiu‐jitsu paradesport practitioners, there are no published data to date. Thus, the objective of this case study was to quantify the magnitude of biochemical, hematological, and urinary alterations after a simulated fight session in elite athlete with world titles. Outcomes were obtained through blood analysis of samples collected at four different moments (M1‐fasting; M2—1.5 hr after caloric intake; M3—Immediately after the simulated fight; M4—24 hr after the simulated fight). Responses triggered by the simulated fight between baseline and after 24 hr were found to increase in monocyte (100%), neutrophil (20%), and insulin (57%) concentrations, while reductions were observed in eosinophils (−50%), lymphocytes (−26.6%), platelets (−22%), cortisol (−50%), and creatine phosphokinase (−45.2%). After 24 hr lactate values returned to baseline levels. The different changes in biochemical and hematological parameters observed constitute responses to acute physical exercise and were according to the level of the high performance athlete. From these data it will be possible to evaluate the periodization, training load, and recovery techniques according to the individual response verified. In addition, these data may be used for comparison purposes within this specific sport, whose literature is still limited.

## Graphical abstract

This study quantified the magnitude of acute biochemical and hematological changes in an amputee elite athlete, practicing para‐sports jiu‐jitsu.

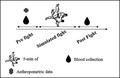

## 1 INTRODUCTION

Paradesport comprises several sports including swimming, rugby, basketball, canoeing, table tennis, soccer, athletics, taewkondo, judo, and jiu‐jitsu, offering practitioners different options. Regarding Brazilian jiu‐jitsu, there is a paradoxical context. On the one hand, its increasing popularity, and on the other, the finding of important gaps in the scientific literature related to this sport (Gonçalves, de Magalhães Neto, & Lopes, 2019; Lopes, de Magalhães Neto, et al., 2019).

The acyclic and intermittent character of the Brazilian jiu‐jitsu paradesport fight has a high intensity and generates high metabolic stress (Lopes, Lopes, & Magalhães Neto, 2019), responsible for acute hematological, hormonal, and biochemical alterations that modulate the immune system in a chronic way (Baumgart, Brurok, & Sandbakk, 2018; Gonçalves et al., 2012; Heyward, Vegter, Groot, & Woude, 2017; Nowak et al., 2017). In this context, data on bioenergetics and metabolism are needed to understand specific physiological changes during combat. This fact should be considered when prescribing physical training strategies, which should be based on parameters presented by specific studies. In this regard, studies with sports methods have emerged in recent decades as an ideal strategy to study the interactions between exercise, the immune system, and metabolism via cellular adaptations (Bassini‐cameron, Bottino, Bittar, Veiga, & Cameron, 2007; Bessa et al., 2008; Coelho et al., 2016).

In this respect, recent studies (Brandão, Fernandes, Alves, Fonseca, & Reis, 2014; Mujika, 2016; Rama, Minuzzi, Carvalho, Costa, & Teixeira, 2016) investigated the behavior of hematological and urinary variables to discuss various parameters related to the recovery profile, effort intensity, stress, fatigue, immunity, and inflammation verified in response to sports practice, in order to characterize the physiological profile in specific modalities. However, as mentioned, there is an important gap regarding investigations implemented in Brazilian jiu‐jitsu, especially for paradesports.

On the other hand, the knowledge of the mentioned parameters helps in understanding the specific needs of the organism, and allows specific prescribed procedures and ergogenic strategies that meet the demands of the combat (Lopes, de Magalhães Neto, et al., 2019). Accordingly, specific markers such as lactate, cortisol, insulin, and urinary pH, provide a basis for diagnosis and allow the suggestion of protocols that help the process of tissue recovery after exertion. In addition, hematological data are able to demonstrate the condition of the immune system, necessary for good levels of fitness and sports performance.

Based on the above, it is believed that conducting case studies that include participants with distinct and particular characteristics, especially in underexplored populations, are important for measurement and discovery of parameters, which have intrinsic potential in the management of future interventions. In this sense, Halperin's study (Halperin, 2018) justified that case studies have much recognized benefits regarding the possibility of using the data as an important tool used by trainers in practical scenarios, and may serve as an agreement strategy between scientists and trainers, creating future opportunities in research collaborations.

To the best of our knowledge, no previous study has investigated the variables presented during a Brazilian jiu‐jitsu paradesport fight. Thus, in the light of the above, this study aimed to investigate for the first time the behavior of blood and urinary markers during a Brazilian jiu‐jitsu paradesport fight in a world champion athlete, with proximal transfemural amputation, in response to a simulated fight.

## 2 METHODS

### 2.1 Subject

The participant included in this case study was a 43‐year‐old male high performance Brazilian Jiu‐Jitsu Paradesport athlete, 1.63 meters of height, body mass of 96.5 kilograms, training time in jiu‐jitsu of 22 years and average weekly training time, corresponding to 18 hr. Regarding the functional classification, the participant was evaluated in class “A”, according to the Brazilian Federation of Brazilian Jiu‐jitsu Paradesport, characterized by proximal transfemural amputation of the left lower limb.

The athlete in question was a world champion five times in a row at the Grand Slam event, held respectively in Brazil, the United States, England, Japan, and Adu Dhabi, all organized by the Brazilian Jiu‐Jitsu Federation (UAEJJF), being one of the pioneers of this modality in Brazilian paradesports.

All objectives and procedures of the case study were presented to the participant who, after agreeing, signed an informed consent form, ensuring his privacy. All procedures were approved by the Human Research Ethics Committee of the Federal University of Mato Grosso, campus of Araguaia. Approval Number: 2.230.073.

### 2.2 Case study design

Data collection was carried out in June 2019 at the participant's usual training center (Grace Barra Academy) located in Barra do Garças—MT, Brazil, and data analyzes were conducted at the Federal University of Mato Grosso, Araguaia Campus. All procedures were performed under standard conditions (temperature: 28 ± 1°C, relative humidity: 84%).

During the case study, the athlete reported no health problems, did not use ergogenic substances or medications. As the participant is an elite athlete, he has a balanced diet routinely controlled by a nutritionist. Additionally, the participant received guidance reinforcing against the excessive consumption of caloric foods and caffeine one day before the experiment. On the day of the experiment, this control was subjectively confirmed.

At first, participant was submitted to an anthropometric evaluation (Table 1) using a weight scale (Tanita BC554, Iron Man/InnerScaner, Tanita) and a stadiometer (Sanny, American Medical do Brasil). The body mass index was calculated as BM divided by the square of the body height (Lopes et al., 2018).

**TABLE 1:** Mean and *SD* values of hematological and biochemical markers of muscle damage during the analyzed moments

	M1	M2	M3	M4	*Chang* (Δ %)
Hematological parameters					
Erythrocytes (µl)	5.2	5.0	5.7	4.8	−7.69
Hemoglobin (g/dl)	15.8	15.3	17.4	17.5	−8.23
Hematocrit (%)	47	46	52	43	−8.51
ACV fl	90.2	91.3	91.7	88.8	−1.55
ACH (pg)	30.3	30.4	30.7	30	−0.99
ACHC (%)	33.6	33.3	33.5	33.7	0.30
Total leukocytes (cells/µl)	6,400	7,200	14,500	7,000	9.38
Neutrophils (cells/µl)	50	52	30	60	20.0
Eosinophils (cells/µl)	2	2	0	1	−50.0
Lymphocytes (cells/µl)	45	42	63	33	−26.67
Monocytes (cells/µl)	3	4	7	6	100
Platelets (cells/µl)	227,000	172,000	267,000	177,000	−22.2
Biochemical parameters					
Lactate (µg/dl)	0.74	1.55	4.58	0.71	−4.05
Cortisol (ug/dl)	8.4	9.7	17.69	4.2	−50.0
Insulin (wm/ml)	16.2	16.1	8.94	25.5	57.41
CPK (U/L)	409	566	362	224	−45.23
LDH (U/L)	174	185	176	163	−6.32
AES density	1,015	1,020	1,010	1,015	0.0
AES pH	5	6	6.6	7	40

Note

Δ%, change from baseline. Fasted baseline (M1), post‐feed (M2), post‐fight (M3), +24 hr (M4)

Abbreviations: ACH, average corpuscular hemoglobin; ACHC, average corpuscular hemoglobin concentration; ACV, average corpuscular volume; AES, abnormal elements of sediment; CPK, creatine phosphokinase; LDH, lactate dehydrogenase; *SD*, standard deviation.

**FIGURE 1:**
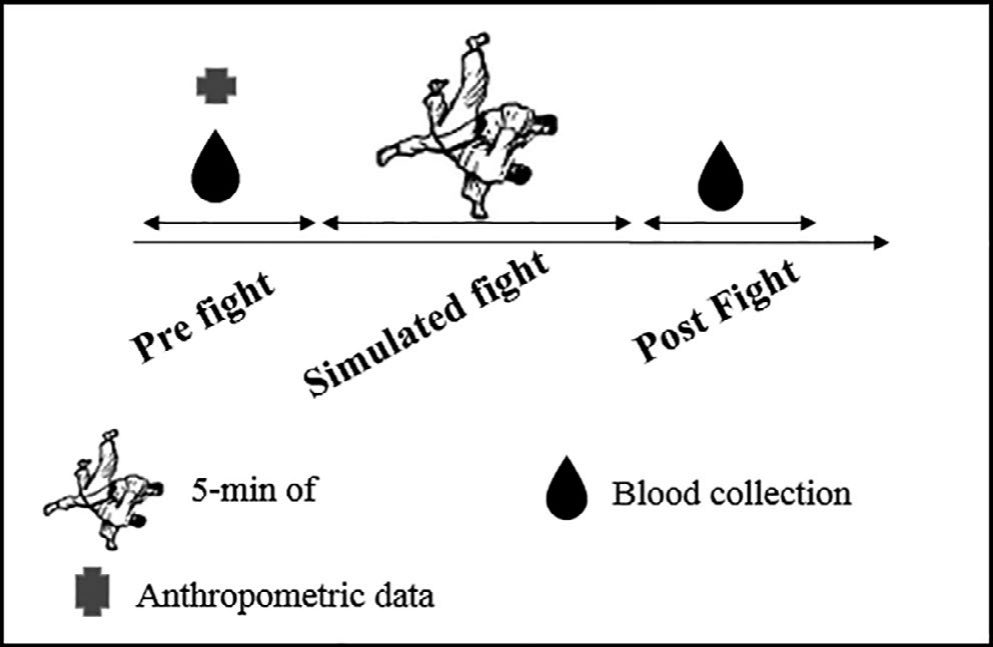
Study design

The biochemical and hematological data determined from blood samples collected at four consecutive moments (M1, M2, M3, and M4) are shown in Table 1. In M1, the participant was fasting for eight hours. The collection in M2 occurred 1 hr and 30 min after the participant ingested a meal (consisting of 200 calories, 10% fat, 77% carbohydrate and 13% protein and 200 ml isotonic drink, 46 calories distributed in 0% fat, 100% carbohydrate, 0% protein). Immediately after the fight, there was a new blood collection, characterizing the M3. Finally, M4 occurred 24 hr after the simulated fight (Figure 1).

### 2.3 Procedures

#### 2.3.1 Simulated fight protocol

All procedures of the fight protocol used in this case study occurred in line with the design suggested by Lopes et al. (Lopes, de Magalhães Neto, et al., 2019). Thus, a warm‐up was performed with Brazilian jujitsu movements of low intensity, characterized by low heart rate and low force requirement, for five minutes. The simulated fight protocol occurred in accordance with the rules of the Brazilian Jiu‐Jitsu Sports Confederation, with six minutes of duration, excluding any type of finalization. In these cases, the athletes were separated and directed to return to the fight immediately. Thus, maximum effort was advocated. In addition, after 3 min of fighting the opponent was replaced by a rested opponent to maintain as high an intensity as possible and to implement the highest possible metabolic stress. The parathlete fought with nondisabled athlete.

The fight protocol began with kneeling athletes to minimize chances of injury by falling. Both subjects were instructed to maintain high mobility and to prevent the fight from finishing before the scheduled time.

#### 2.3.2 Blood sampling and analysis

Blood collection was performed in the antecubital vein by a qualified professional. All blood sampling, centrifugation, separation, and storage procedures were performed immediately after blood collection to preserve sample integrity.

The following hematological parameters were quantified: erythrogram (hematocrit, erythrocytes, and hemoglobin) leukogram (total leukocytes, monocytes, neutrophils, lymphocytes), platelets, cortisol, insulin, creatine phosphokinase (CPK), lactate dehydrogenase (LDH), and blood lactate.

Enzymatic method was used for the measurement of serum levels of CPK (IU/L), blood lactate (IU/L), and LDH (IU/L) using a semiautomatic Semi‐auto biochemical analyzer (BC‐300, Qinhuangdao).

Samples for the biochemical assay were collected in tubes with a coagulation intensifier and split gel (Vacuette, Greiner Bio‐One) and immediately centrifuged (3,000 rpm/10 min). Blood serum was aliquoted and stored in liquid nitrogen for future analysis using specific kits and according to the manufacturer's specifications (ELISA Kit), for analyzes of cortisol and insulin. In turn, data from complete analysis of erythrogram, leukogram, and platelets was measured using the Sysmex XE‐5000 hematological analyzer, according to all specifications recommended by the manufacturer. Blood collection and analysis was based on the methodology used in the study by Lopes, de Magalhães Neto, et al. (2019)).

#### 2.3.3 Urine sampling and analysis

The urine was collected in an appropriate sterile container (Brand: MYLABOR/Model: UNIVERSAL COLLECTOR) supplied to the athlete. Thus, the analysis of abnormal sediment elements was performed in two parts. The first was performed by chemical reactions and the second by visualization of urine drops under the microscope (Olympus, cx21 fs), observed in the 100‐fold increase. To prepare the urine sample for microscopic analysis, a fresh sample of 10 to 15ml of urine was centrifuged at 1,500 to 2,000 rpm for 10 min (EBA 270). The method of analysis used was based on the model suggested the study by Liang et al. (Liang et al., 2018).

### 2.4 Data presentation

To facilitate visualization and comparison, some data were normalized to the M1 measurement of each trial (absolute values are referenced in each figure). All other data are given as an absolute value. The data obtained were presented as standardized graphs (relative) made using the Sigmaplot program. Comparisons of the variables analyzed between basal and post fight protocol moments were calculated by the mean of the differences in percentage. Correlation analyzes were verified using the CorReg program, Version: 1.0 in order to identify a possible association between investigated markers.

## 3 RESULTS

Table 1 presents data related to the markers evaluated at the described moments, together with the Chang coefficient representing the percentage change values from the baseline.

### 3.1 Hematological parameters

**FIGURE 2:**
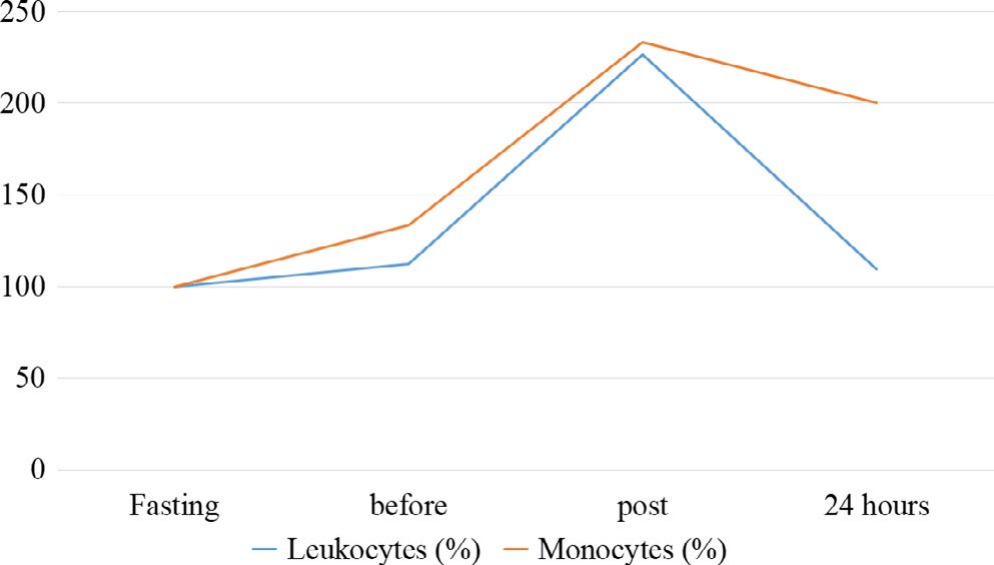
Behavior of hematological parameters during the moments evaluated in the case study. Normalized values (%) for baseline results

**FIGURE 3:**
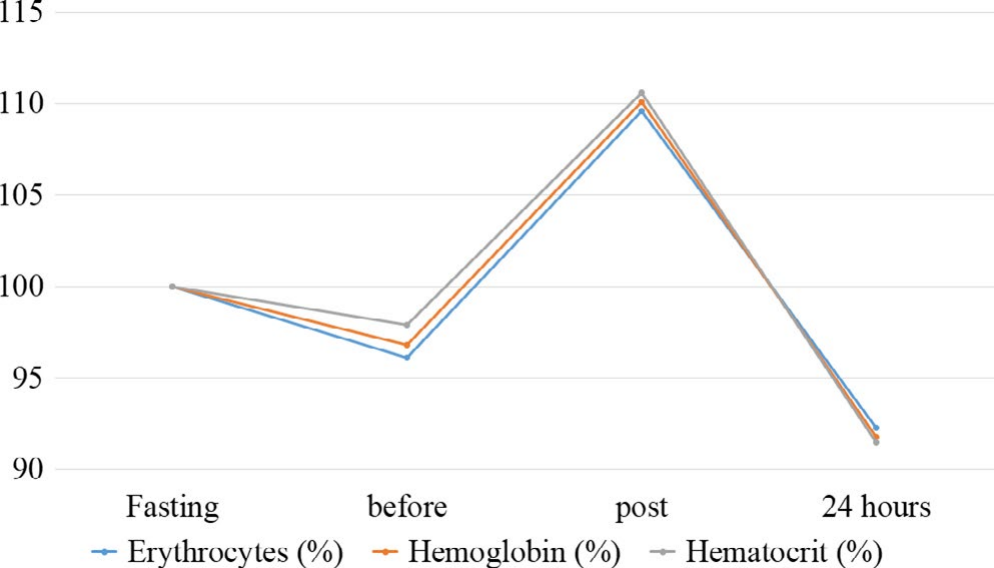
Behavior of erythrocytes, hemoglobin, and hematocrit during the moments evaluated in the case study. Normalized values (%) for baseline results

**FIGURE 4:**
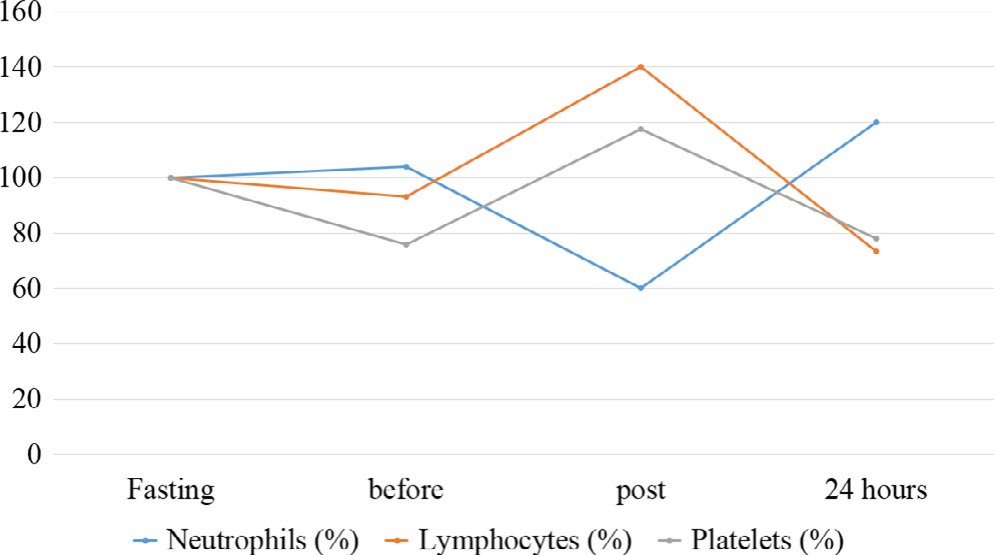
Behavior of neutrophils, lymphocytes, and platelets during the moments evaluated in the case study. Normalized values (%) for baseline results

The outcomes of hematological parameters are presented in Table 1 and Figures 2, 3, 4.

Regarding the results corresponding to the erythrogram, similar behavior of erythrocytes, hemoglobin, and hematocrit was observed. Thus, a slight reduction was observed in the analytical cited between fasting and prefight moment, with subsequent increase after the fight, which reduced to lower values than the basal, in 24 hr. *Chang* values for the variation observed for such parameters corresponded to 7.69%, 8.23%, 8.61% respectively. In addition, there was a high correlation (*r* = .99) between these analytics.

The findings regarding total leukocytes and monocytes showed similar behavior at baseline, pre‐ and postfight. However, at 24 hr the monocytes remained at a relative value at twice the baseline (100%) whereas leukocytes approached the recovery from baseline (9.38%). About these data, a correlation of .76 was verified.

Regarding lymphocytes and platelets, similar kinetic behavior was verified, which showed inversely proportional values, at all analyzed moments, correlated with neutrophil values. The variation verified between the initial and final moment for these markers was −26.6% for lymphocytes and −22.2% for platelets. However, at 24 hr they were lower than those observed at baseline. In contrast, the neutrophil variation was 20%. The correlation between these registered parameters, characterized value corresponding to 0.89.

### 3.2 Metabolic and urinary parameters

**FIGURE 5:**
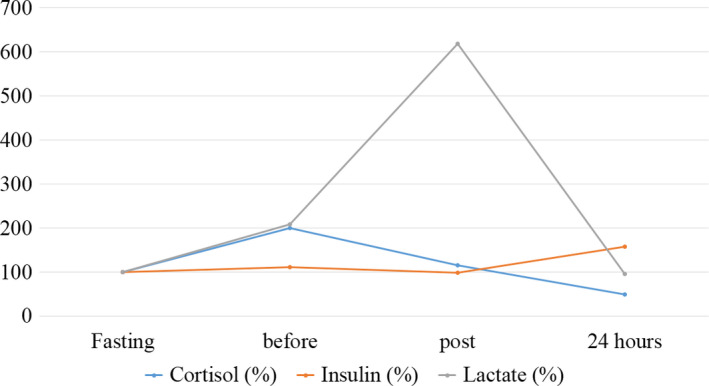
Behavior of cortisol, insulin, and lactate parameters during the moments evaluated in the case study. Normalized values (%) for baseline results

**FIGURE 6:**
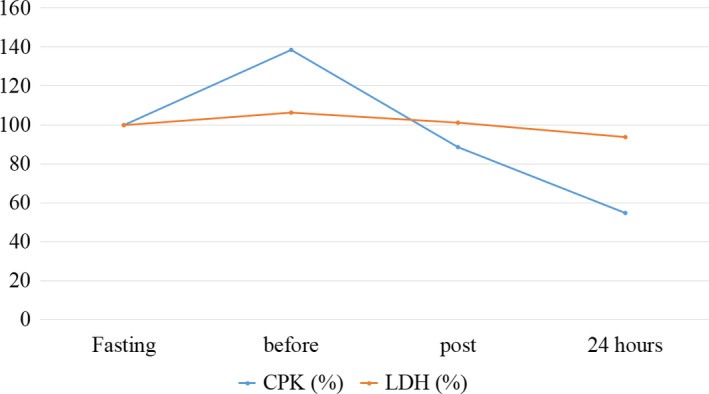
Behavior of CPK and LDH parameters during the moments evaluated in the case study. Normalized values (%) for baseline results. CPK, creatine phosphokinase; LDH, lactate dehydrogenase

Metabolic parameters were investigated to obtain information regarding exercise intensity and muscle damage. The data presented below are also shown in Table 1 and Figures 5 and 6.

Lactate behavior analysis showed an increase of 600% in the postfight moment, with a 24‐hr reestablishment of the baseline value, characterizing a −4.05% variation between the basal and final moments.

The cortisol and insulin hormones presented antagonistic profile, with inverse responses in all analyzed moments. Moreover, within 24 hr, the values of both did not return to baseline, with variation values corresponding to −50.0% for cortisol and 57.2% for insulin. The correlation between insulin and cortisol was .97.

Regarding the CPK and LDH enzymes, similar behaviors were found, however, with analytical levels in very different degrees. Regarding the variation between baseline and 24 hr, it was found that at the end, both enzymes showed lower values than initially observed, equivalent to −45.23% for CPK and −6.32% for LDH.

Finally, results regarding urine density expressed homogeneous and unchanged behavior during the verified moments, with *Chang* equal to 0%. In contrast, urinary pH showed increasing values, which did not recover within 24 hr (*Chang* equals 40%).

## 4 DISCUSSION

This case study aimed to identify changes in biochemical, hematological, and urinary parameters after a simulated Brazilian Jiu‐jitsu Paradesport fight. The main findings demonstrated a considerable increase in all postfight hematological parameters investigated, followed by a subsequent reduction in 24 hr, with recovery close to baseline levels. The metabolic and urinary parameters, on the other hand, demonstrated particular responses for each investigated variable, which will be discussed below.

Regarding total leukocytes, the monocyte type showed similar behavior in the present case study, with values that increased in the postfight moment but reduced in the 24‐hr moment. Recent studies (Coswig, Neves, & Del Vecchio, 2013; Coswig, Neves, & Del, 2013) investigated the same variables, in a similar scenario, with elite Brazilian jiu‐jitsu athletes, finding similar outcomes to ours with paradesport athlete. These data relate to the presence of transient inflammatory response observed after intense exertion in which there is alteration in innate immune response (represented by natural killer cells and leukocytes) as well as to the acquired immune system (antibodies), but which returns to basal levels within a few hours after the effort (Lopes, de Magalhães Neto, et al., 2019). In conclusion, these results demonstrate that there appears to be no difference between the acute immune response induced by Brazilian jiu‐jitsu practice between disabled and nondisabled athletes.

The results of this case study showed that lactate increased 600% after the fight compared to baseline, with almost full recovery within 24 hr. These data agree with other studies that report an increase in this metabolite after intense exercise, being a commonly used predictor for effort intensity measurement (Belli et al., 2018; Damas et al., 2016; Liang et al., 2018). Lactate is an intermediate of carbohydrate metabolism in anaerobic condition, as O_2_ consumption does not increase instantaneously and proportionally to the initial energy demand of exercise. Thus, the bioenergetic pathways for ATP production during high intensity exercise are muscle glycogenolysis and glycolysis, culminating with the increase in lactate production, which is consistent with the result observed after the fight. However, part of the lactate produced in skeletal muscle is captured by liver cells and converted into glucose through gluconeogenesis, an important metabolic pathway for maintaining blood glucose, which justifies the decrease in plasma lactate levels after 24 hr (Adeva‐Andany et al., 2014; Papassotiriou & Nifli, 2018).

In addition, the normalization of blood lactate also suggests that this outcome may be extrapolated to conclusions related to skeletal muscle tissue recovery, allowing to infer that the sport is being performed at safe intensity levels (Adeva, González‐Lucán, Seco, & Donapetry, 2013), minimizing the occurrence of chronic damage to the athlete body systems (Lopes, de Magalhães Neto, et al., 2019). The evaluation of plasma LDH activity corroborates this hypothesis, since it is a cytosolic enzyme and is responsible for the reversible reduction of pyruvate to lactate. Elevated plasma levels indicate tissue damage and cell lysis; however, no increase in plasma activity was observed immediately and 24 hr after exercise.

Regarding cortisol levels, studies show that the increase of this marker in the described conditions, in simulated fight, indicates increased catabolite status (Greenham, Buckley, Garrett, Eston, & Norton, 2018; Liang et al., 2018). Cortisol is the major glucocorticoid secreted by the adrenal cortex. Intense exercise induces the release of adrenocorticotropic hormone by the anterior pituitary, which binds to receptors in the adrenal cortex and increases cortisol secretion. Cortisol‐mediated signaling pathways are related to controlling the availability of energetic substrates (glucose and fatty acids), as well as stimulating proteolysis to release amino acids used in tissue repair. However, cortisol values decreased below baseline levels within 24 hr, which apparently suggests adequate adaptive responses and postexercise recovery system.

Insulin is secreted by β‐pancreatic cells and is the main hormone involved in glucose absorption and storage. Plasma insulin concentration decreases during exercise with increasing intensity in order to maintain glycemic concentration and appropriate levels. In addition, acute and chronic exercise induces a change in the number of membrane glucose transporters, which increases insulin sensitivity, reducing insulinemia. Andreato et al (Andreato et al., 2015) reported decreased insulin after exertion in response to catabolic activity.

Creatine phosphokinase enzyme consists of a dimer composed of two subunits (B and M) and has three isoforms: CK‐BB or CK‐1 found mainly in the brain; CK‐MB or CK‐2, hybrid form, mainly in the myocardium and CK‐MM or CK‐3 mainly in skeletal muscle. The reaction catalyzed by CPK is to reversibly phosphorylate creatine at the expense of ATP with the creation of creatine phosphate, especially at rest, so that during muscle activity, the reaction occurs in reverse, aiming at the synthesis of ATP (Miyamoto et al., 2018). When the intensity of the exercise is adequate to the metabolic capacity of the muscle tissue, there is little change in the membrane permeability of the muscle cell without altering the plasma concentration of the enzyme. However, if the effort intensity exceeds this capacity, the membrane permeability increases and the CPK enzyme is released from the intracellular environment to the circulation, which characterizes muscle damage (Simpson & Hager, 1984). The results show a reduction in serum CPK levels indicating again that the participant's adaptive response is appropriate to the intensity of the exercise performed.

High intensity physical activity also leads to increased production of hydrogen ions sufficient to cause significant reduction in muscle and plasma pH, negatively influencing athlete performance (Greenham et al., 2018). However, the body has systems capable of regulating acid‐base balance and preventing sudden changes in pH. The main buffering systems for exercise‐induced hydrogen ion production are intracellular proteins, bicarbonate buffer, and phosphate buffer, in addition to the increased pulmonary ventilation during intense exercise, which favors the elimination of carbon dioxide by controlling acidosis (Simpson & Hager, 1984). The renal contribution to the regulation of acidic balance during exercise is irrelevant due to the time required for the response. The kidneys take several hours to respond effectively to the increase in the concentration of hydrogen ions and this explains the result observed in this case study where urinary pH did not return to baseline even after 24 hr of the fight.

These findings are important in a practical setting (Bassini & Cameron, 2014), providing trainers and physical therapists with specific physiological parameters triggered in response to the practice of Brazilian Jiu‐jitsu Paradesport, which allows periodization and implementation of specific recovery techniques (Machado et al., 2018), based on the presented outcomes. However, some limitations need to be reported. In this regard, long‐term follow‐up has not been performed, so further studies are needed to characterize the long‐term physiological effects and adaptations triggered in response to combat in the investigated markers. It is important that future studies with high methodological quality, such as randomized clinical trials, investigate the effects of specific recovery techniques, on this athletic profile. It is reiterated that the data presented should not be extrapolated to different population profiles.

Finally, we emphasize that the data presented in this case study were obtained from procedures performed in the field, identical to those used in competitive combat, are unpublished and constitute potential under new perspectives and parameters related to Brazilian Jiu‐Jitsu Paradesport. Thus, we believe that these results can facilitate trainers and athletes a better understanding of the demands of paradesport, helping to monitor adaptation and sports performance based on individualized training plans.

## 5 CONCLUSION

The simulated fight of Brazilian Jiu‐jitsu Paradesport is responsible for triggering changes in the body's homeostasis in hematological and biochemical parameters and the observed modifications constitute acute responses to intense physical exercise. Monitoring these variables allows the adjustment of periodization and recovery techniques for the sport in question. Additionally, these data can be used to prevent injuries and incorporate changes in training load according to individual responses.

## AUTHOR CONTRIBUTIONS

AMMN and CMBA designed the case study, conducted the analyses. JSSL wrote the manuscript, assisted in the acquisition, analysis, and interpretation of data, and reviewed and edited the article. ACA, ELS, MVAV, and PRLA performed a critical revision of the article. All authors read and approved the final manuscript.
